# LncRNA KCNQ1OT1 promotes the metastasis of ovarian cancer by increasing the methylation of *EIF2B5* promoter

**DOI:** 10.1186/s10020-022-00521-5

**Published:** 2022-09-13

**Authors:** Si-Li He, Ya-Ling Chen, Qi-Hua Chen, Qi Tian, Shui-Jing Yi

**Affiliations:** 1 Department of Gynaecology, The Third Xiangya Hospital of Central South University, No. 138, Tongzipo Road, Changsha, 410013 Hunan Province People’s Republic of China; 2 Hunan University of Chinese Medicine, Changsha, 410013 Hunan Province People’s Republic of China; 3 Surgical Department, The First Hospital of Hunan University of Chinese Medicine, Changsha, 410017 Hunan Province People’s Republic of China; 4 Department of Medical Genetics, Maternal and Child Health Hospital of Hunan Province, Changsha, 410013 Hunan Province People’s Republic of China

**Keywords:** Ovarian cancer, lncRNA KCNQ1OT1, EIF2B5, Methylation, Proliferation, Metastasis, Invasion

## Abstract

**Background:**

Long non-coding RNAs (lncRNAs) have emerged as regulators of human malignancies, including ovarian cancer (OC). LncRNA KCNQ1OT1 could promote OC progression, and EIF2B5 was associated with development of several tumors. This project was aimed to explore the role of lncRNA KCNQ1OT1 in OC development, as well as the involving action mechanism.

**Methods:**

Reverse transcription quantitative polymerase chain reaction (RT-qPCR) or Western blotting was employed to determine the expression levels of KCNQ1OT1 and EIF2B5. OC cell proliferation was evaluated by MTT and colony formation assays, and wound healing and Transwell assays were implemented to monitor cell migration and invasion, respectively. The methylation status of EIF2B5 promoter was examined by MS-PCR, to clarify whether the expression of EIF2B5 was decreased. The binding activity of KCNQ1OT1 to methyltransferases DNMT1, DNMT3A and DNMT3B was determined by dual luciferase reporter assay or RIP assay, to explore the potential of KCNQ1OT1 alters the expression of its downstream gene. ChIP assay was carried out to verify the combination between EIF2B5 promoter and above three methyltransferases.

**Results:**

Expression of lncRNA KCNQ1OT1 was increased in OC tissues and cells. EIF2B5 expression was downregulated in OC, which was inversely correlated with KCNQ1OT1. Knockdown of KCNQ1OT1 inhibited OC cell proliferation and metastasis. KCNQ1OT1 could downregulate EIF2B5 expression by recruiting DNA methyltransferases into EIF2B5 promoter. Furthermore, interference of EIF2B5 expression rescued KCNQ1OT1 depletion-induced inhibitory impact on OC cell proliferation and metastasis.

**Conclusion:**

Our findings evidenced that lncRNA KCNQ1OT1 aggravated ovarian cancer metastasis by decreasing EIF2B5 expression level, and provided a novel therapeutic strategy for OC.

## Highlights

1. LncRNA KCNQ1OT1 is upregulated, while EIF2B5 is downregulated in OC tissues and cells.
2. Knockdown of KCNQ1OT1 represses OC cell proliferation and metastasis.
3. KCNQ1OT1 decreases EIF2B5 expression by recruiting DNA methyltransferases into EIF2B5 promoter, thereby promoting OC progression.

## Introduction

Ovarian cancer (OC) is one of the most prevailing tumors derived from reproductive system in women, and ranks as the most lethiferous gynecological malignancy (Szajnik et al. 2016). Due to lacking obvious symptom at early stage, majority of OC patients were diagnosed at advanced stages, whose 5-year survival rate is about 44% worldwide (Gilbert et al. 2012; Siegel et al. 2019). Though current therapeutic tactics, like surgery, immunotherapy and targeted therapies, have worked much. Overall prognosis of OC patients is not optimistic, and recurrence post treatment is prone (Odunsi 2017; Hennessy et al. 2009). Hence, further investigation on the molecular mechanism of OC occurrence and progression might be beneficial to clinical therapy.

LncRNAs are non-coding RNAs with more than 200 nucleotides long, that involved in multiple cellular processes, such as cell survival, proliferation, mobility and differentiation (Peng et al. 2016). With the improvement of sequencing technology, numerous lncRNAs were discovered, and certain dysregulated lncRNAs were manifested to be associated with OC progression (Zhong et al. 2016). LncRNA KCNQ1 opposite strand/antisense transcript 1 (KCNQ1OT1) was a well-established promoting factor of OC growth and metastasis (Liu et al. 2020). Additionally, a recent study reported that KCNQ1OT1 participated in Yin Yang 1 (YY1)-mediated tumor-promoting effects on Triple-negative breast cancer (TNBC) through its interactions with DNMT1, in support of role of KCNQ1OT1 in TNBC (Shen et al. 2020). However, the action mechanism by which KCNQ1OT1 exerted roles in OC has not been fully uncovered.

Eukaryotic translation initiation factor 2B (EIF2B) is essential for mRNA translation containing five subunits, of which EIF2Bε was coded by EIF2B5 (Wortham and Proud 2015). In addition, dysregulated EIF2B5 expression was also corrected with the progression of liver cancer (Jiao et al. 2018), colorectal cancer (Palaniappan et al. 2016) and diffuse large B-cell lymphoma (Unterluggauer et al. 2018). EIF2B5 was lowly expressed in OC tissues, implying its relevance with OC (Hou et al. 2020). MethPrimer 2.0 predicted that there were CpG islands in EIF2B5 promoter region, suggesting that EIF2B5 methylation was increased. Former research indicated that lncRNA could affect DNA methylation, so as to reduce the expression of gene (Fang et al. 2016). Therefore, we speculated that KCNQ1OT1 might alter EIF2B5 expression by affecting methylation.

In this work, we detected the upregulation of lncRNA KCNQ1OT1 and the downregulation of EIF2B5 in OC tissues and OC A2780, Anglne, SKOV3 and SW626 cells. Moreover, KCNQ1OT1 could decrease EIF2B5 expression by recruiting DNA methyltransferases into EIF2B5 promoter, thereby facilitating OC development and metastasis.

## Materials and methods

### Collection of clinical tissues

Thirty-two pairs of human tissue samples, including tumor tissues and adjacent healthy tissues, were acquired during surgery at the Third Xiangya Hospital of Central South University, then immediately preserved at − 80 °C. All tissues were confirmed by pathological diagnosis. Before tissue collection, we had got permission from the Ethics Committees of the Third Xiangya Hospital of Central South University and informed consent from all these 32 OC patients. None of OC patients had received preoperative therapy. Clinicopathological characteristics of all OC patients were listed in Table 1.

**Table 1:** Clinical characteristics of OC patients with high and KCNQ1OT1 risk scores

	Case (n)	KCNQ1OT1		P
		Low-risk (n)	High-risk (n)	
Age				
≥ mean	20	12	8	1.0
< mean	12	7	5	
FIGO stage				
I–II	22	18	4	0.0369
III–IV	10	4	6	
Histological type				
I	5	4	1	0.0367
II–III	27	7	20	
Residual tumor (cm)				
< 1 cm	20	10	10	0.7257
≥ 1 cm	12	7	5	
Histology				
Mucinous	5	3	2	1.0
Setous	16	10	6	
Endometrioid	7	4	3	
Clear cell	4	2	2	
Lymphatic metastasis				
No	18	8	10	0.7224
Yes	14	8	6	
Ascites				
No	15	9	6	1.0
Yes	17	10	7	

### Cell culture and transfection

Human ovarian surface epithelial cells (IOSE-80#, LMAIBio, Shanghai, China), OC A2780 cells (CL-0013#, Procell, Wuhan, China), Anglne (CL-0024#, Procell), SKOV3 (HTB-77#, ATCC, Manassas, VA, USA), SW626 (HTB-78#, ATCC), COV362 (07071910#, ECACC, European Collection of Authenticated Cell Cultures), CAOV3 (HTB-753#, ATCC) and OVCAR-3 (HTB-161#, ATCC) cells were cultured in a humidified incubator with 5% CO_2_ at 37 °C. Cells were maintained in RPMI-1640 medium (Sigma-Aldrich, St. Louis, MO, USA) mixed with 10% FBS (Gibco, Gran Island, NY, USA) and 1% penicillin–streptomycin (Sigma-Aldrich). Lentiviral-based shRNA targeting KCNQ1OT1 (sh-KCNQ1OT1; GenePharma, Shanghai, China) or EIF2B5 (sh-EIF2B5; GenePharma) was stably transfected into SKOV3 and SW626 cells to silence KCNQ1OT1 or EIF2B5 expression, with sh-NC (GenePharma) as negative control.

### Reverse transcription quantitative polymerase chain reaction (RT-qPCR)

Total RNA of clinical tissues or cells was extracted using TRIzol Reagent (Invitrogen, Vienna, Austria), then RNA quality was determined using NanoDrop ND-1000 spectrophotometer (NanoDrop Technologies, Wilmington, DE, USA). Then cDNA was synthesized with 1 μg RNA template and M-MLV Reverse Transcriptase (Invitrogen). RT-qPCR was performed using SYBR Green PCR Kit (Qiagen, Frankfurt, Germany) to evaluate the expression of KCNQ1OT1, EIF2B5, DNMT1, DNMT3A and DNMT3B, with GAPDH as internal control. Relative expression was analyzed using 2^−ΔΔCt^ method (Livak and Schmittgen 2001). All RT-qPCR primers were synthesized by Sangon Biotech (Shanghai, China), and primer sequence was shown in Table 2.

**Table 2:** The primer sequence for RT-qPCR assay

Gene	Sequence	
	Forward (5′–3′)	Reverse (5′–3′)
KCNQ1OT1	TTGGTAGGATTTTGTTGAGG	CAACCTTCCCCTACTACC
EIF2B5	TCTGGCACTGTCATTGGCAGCA	GAGACTGATGGATCTGTGCTCC
DNMT1	AGGTGGAGAGTTATGACGAGGC	GGTAGAATGCCTGATGGTCTGC
DNMT3A	CCTCTTCGTTGGAGGAATGTGC	GTTTCCGCACATGAGCACCTCA
DNMT3B	TAACAACGGCAAAGACCGAGGG	TCCTGCCACAAGACAAACAGCC
GAPDH	CCATGTTCGTCATGGTGTG	GGTGCTAAGCAGTTGGTGGTG

### Western blotting

Clinical tissues or cells were lysed in RIPA buffer (Beyotime, Nantong, China) to extract protein samples, and a BCA Kit (Beyotime) was used to determine protein concentration. Then, 40 μg protein samples were loaded onto 12% fresh SDS-PAGE and transferred to PVDF membranes (Merck Millipore, Darmstadt, Germany). Membranes were blocked with 5% non-fat milk and incubated with rabbit primary antibody anti-EIF2B5 (ab181033; Abcam, Shanghai, China), anti-E-cadherin (ab40772; Abcam), anti-N-cadherin (ab76011; Abcam), anti-DNMT1 (ab188453; Abcam), anti-DNMT3A (ab2850; Abcam), anti-DNMT3B (ab2851; Abcam) or Loading Control anti-GAPDH (ab199554; Abcam), then probed with secondary antibody (ab205718; Abcam). Signals were detected using ECL detection kit (Millipore), and analyzed by Image J software (NIH, Bethesda, MD, USA).

### Cell viability assessment

After transfection, 3 × 10^3^ SKOV3 and SW626 cells seeded in 96-well plates were incubated with 20 μL MTT (Beyotime) for additional 4 h. After removing medium, DMSO was added to dissolve the formazan. 10 min later, the absorbance at 570 nm was recorded using a microplate reader (Bio-Rad, Hercules, CA, USA).

### Colony formation assay

600 transfected SKOV3 and SW626 cells were placed in 6-well plates and continually cultured for 2 weeks at 37 °C. Visible colonies were fixed and stained with 4% paraformaldehyde (Sigma-Aldrich) and crystal violet (Beyotime), respectively. Then, colonies were counted utilizing Image J software.

### Cell migration and invasion assay

OC cells are prone to migrate and invade into the peritoneum and underlying organs (Zhang and Zou 2015). Here, cell migration was examined by wound healing assay. After transfection, SKOV3 and SW626 cells were maintained in 6-well plates containing FBS-free RPMI-1640 medium until confluence reached 80%. A sterile pipette tip (10 μL; Corning Inc., Corning, NY, USA) was applied to create a scratch through the monolayer. Then, PBS was used to wash away detached cells, and cell migration was monitored at same position using a microscope (Nikon, Tokyo, Japan) at 0 h and 24 h after scratch-making.

Transwell chambers (Corning Inc.) pre-enveloped with Matrigel (BD Bioscience, San Jose, CA, USA) were utilized to detect cell invasion. Transfected SKOV3 and SW626 cells suspended in FBS-free medium were transferred to upper chambers. Additionally, complete medium was added to lower ones. 24 h later, invaded cells were fixed with 4% paraformaldehyde, stained with crystal violet and counted under a microscope.

### Nuclear-cytoplasmic fractionation

The current assay combined with RT-qPCR assay were applied to investigate the subcellular location of lncRNA KCNQ1OT1 in OC cells. Firstly, cytoplasmic and nuclear RNA derived from SKOV3 and SW626 cells were separated using a Purification Kit (Norgen Biotek, Thorold, Canada) as instructed by the supplier. Furthermore, GAPDH and U6 snRNA served as internal control for cytoplasmic and nuclear fractions, respectively.

### Fluorescence in situ hybridization (FISH) analysis

FISH assay was executed to determine the cellular localization of in lncRNA KCNQ1OT1 in OC cells. The oligonucleotide probe for KCNQ1OT1 was supplied by Biosense (Guangzhou, China). After mixture with probe mixture (10 μL, Biosense) in the dark overnight, cell nuclei of SKOV3 and SW626 was dyed with 4,6-diamidino-2-phenylindole (DAPI; Beyotime) and visualized under a fluorescence microscope (Olympus).

### MS-PCR assay

This assay was executed to detect the methylation status of the EIF2B5 promoter referring to a former research (Real et al. 2018). The genomic DNA of SKOV3 and SW626 cells was isolated using a commercial kit (TIANGEN, Beijing, China), and a DNA Methylation-Gold™ kit (D5005, Zymo Research, Irvine, CA, USA) was exploited to assess the methylation level of EIF2B5 promoter. Methylated (M) and unmethylated (U) primers were: EIF2B5-M-forward, 5′-GTGATAATGTTGAGGTTAAGGAAC-3′ and EIF2B5-M-reverse, 5′-AAACAACGTAATATTTAAACCCACG-3′; EIF2B5-U-forward, 5′-TGATAATGTTGAGGTTAAGGAAT-3′ and EIF2B5-U-reverse, 5′-AACAACATAATATTTAAACCCACAACC-3′. PCR reaction products were then detected by agarose gel electrophoresis, then images were collected and analyzed.

### RIP assay

The combing potency of KCNQ1OT1 to DNMT1, DNMT3A and DNMT3B was estimated by RPIseq database (http://pridb.gdcb.iastate.edu/RPISeq/). Then RIP assay was carried out to demonstrate the binding potential of KCNQ1OT1 to DNMT1, DNMT3A and DNMT3B proteins. SKOV3 and SW626 cells were lysed in lysis buffer (Beyotime), then generated cell lysate was incubated with the mixture of magnetic beads re-suspended in RIP Wash Buffer (Merck Millipore) and rabbit anti-DNMT1, anti-DNMT3A, anti-DNMT3B or NC anti-IgG (ab133470; Abcam) at 4 °C overnight. After treatment with Proteinase K (Invitrogen), RNA levels were evaluated via RT-qPCR assay.

### ChIP assay

Until cell confluence reached 80%, SKOV3 and SW626 cells were fixed with 1% formaldehyde for 10 min, allowing intracellular DNA and protein to crosslink, followed by ultrasonic treatment. After centrifugation (13,000 rpm, at 4 °C), generated supernatant was incubated with anti-DNMT1, anti-DNMT3A and anti-DNMT3B, or incubated with negative control anti-IgG at 4 °C overnight. Then endogenous DNA–protein complex was precipitated as previously described (Chen et al. 2019). And enrichment of EIF2B5 promoter fragment binding to DNMT1, DNMT3A, and DNMT3B was analyzed by RT-qPCR assay with specific primers for promoter region of EIF2B5 gene: forward, 5′-CTTTCCATGTTTCGCCATCT-3′ and reverse, 5′-AAAGTCCACTGGCACCAAAC-3′.

### Statistical analysis

All assays in this work were performed ≥ 3 times. Data analysis was implemented by SPSS 21.0 software and exhibited as mean ± SD. Student’s *t*-test or ANOVA was applied to compare differences in groups. The correction between levels of KCNQ1OT1 and EIF2B5 in OC tissues was evaluated by Spearman’s correlation coefficient. *P* < 0.05 meant statistically significant.

## Results

### Increased expression of lncRNA KCNQ1OT1 and decreased expression EIF2B5 in OC

**Fig. 1:**
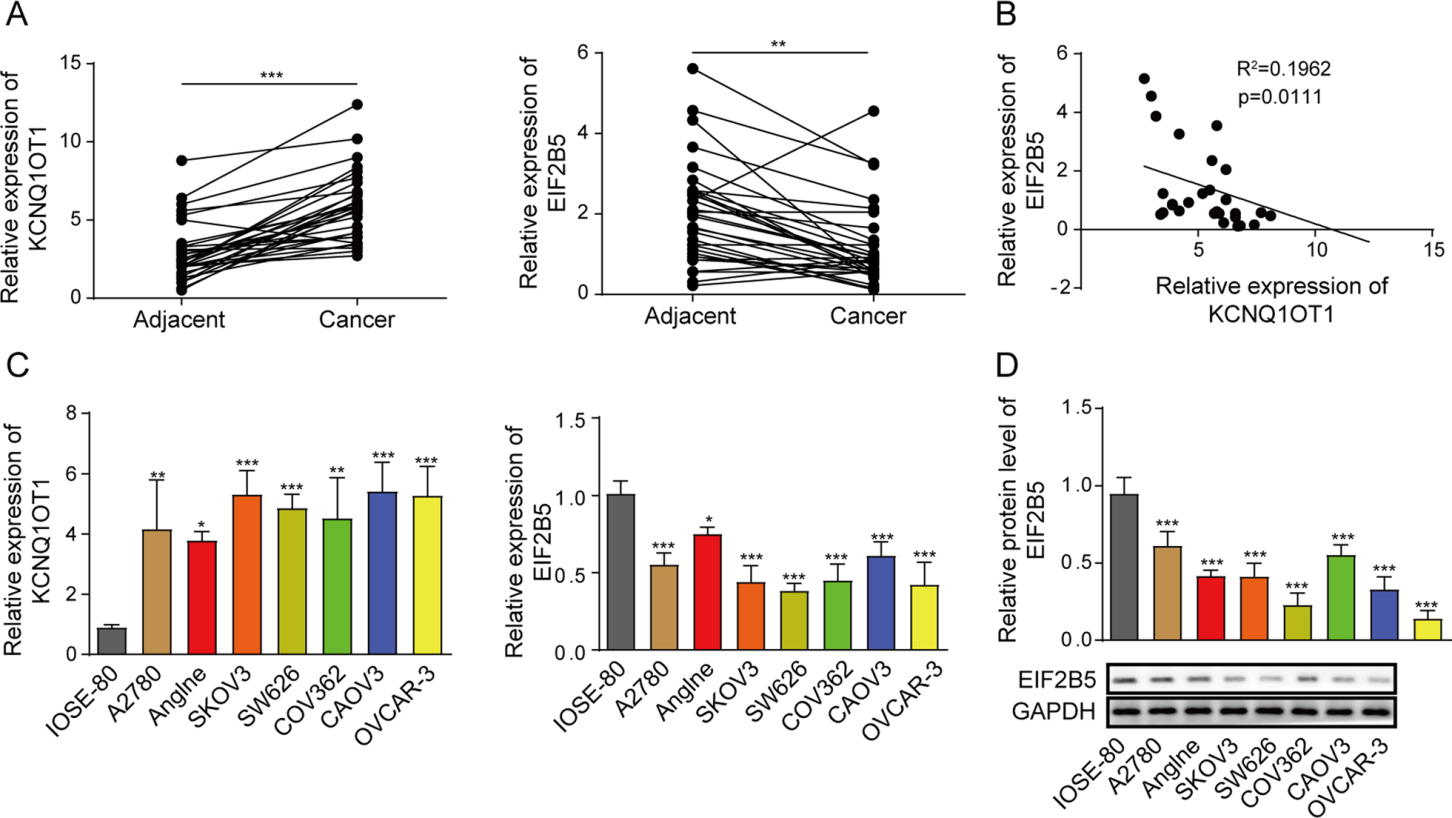
Increased expression of lncRNA KCNQ1OT1 and decreased expression of EIF2B5 in OC. **A** RT-qPCR analysis for the enrichment of KCNQ1OT1 and EIF2B5 in OC tissues and normal tissues (N = 32). **B** Spearman’s correlation analysis for the correlation between expression of KCNQ1OT1 and EIF2B5 in OC tissues (N = 32). **C** RT-qPCR analysis for the enrichment of KCNQ1OT1 and EIF2B5 in IOSE-80, A2780, Anglne, SKOV3 and SW626 cells. **D** Western blotting for abundance of EIF2B5 protein in IOSE-80, A2780, Anglne, SKOV3 and SW626 cells. ***P < 0.001. **P < 0.01. *P < 0.05

Initially, we assessed expression of lncRNA KCNQ1OT1 and EIF2B5 in 32 pairs of OC tissues and normal tissues via RT-qPCR assay. Data demonstrated that KCNQ1OT1 expression was increased in OC tissues relative to normal tissues, while EIF2B5 was lowly expressed (Fig. 1A). Spearman’s correlation analysis disclosed the negative correlation between expression of KCNQ1OT1 and EIF2B5 in OC tissues (R^2^ = 0.1962) (Fig. 1B). Though this finding was inconsistent with TCGA dataset, which might be associated with locality and tissue specificity of KCNQ1OT1 and EIF2B5. Additionally, the upregulation of KCNQ1OT1 and the downregulation of EIF2B5 were detected in A2780, Anglne, SKOV3 and SW626 cells when compared with IOSE-80 cells (Fig. 1C, D). Above results indicated that KCNQ1OT1 was upregulated, while EIF2B5 was downregulated in OC.

### KCNQ1OT1 depletion suppressed the malignant behaviors of OC cells

**Fig. 2:**
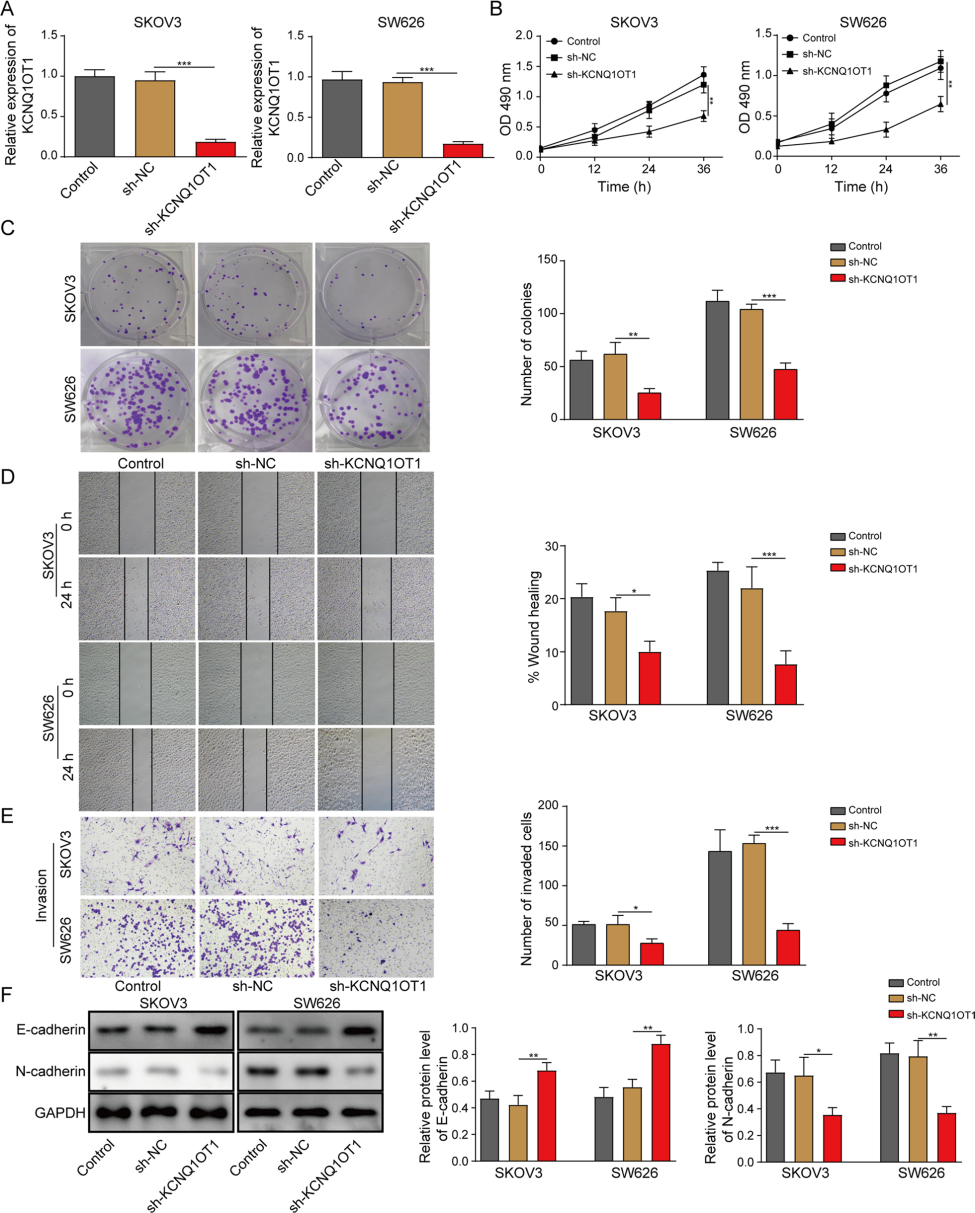
KCNQ1OT1 depletion suppressed the malignant behaviors of OC cells. SKOV3 and SW626 cells were transfected with Control (blank), sh-NC or sh-KCNQ1OT1. **A** RT-qPCR analysis for the enrichment of KCNQ1OT1 in transfected cells. **B** MTT assay for the cell viability of transfected cells. **C** Colony formation assay for the cell clonogenicity capacity of transfected cells. **D** Wound healing assay for the cell migration of transfected cells. **E** Transwell assay for the cell invasion of transfected cells. **F** Western blotting for abundance of E-cadherin and N-cadherin proteins in transfected cells. *P < 0.05

The dysregulation of KCNQ1OT1 encouraged us to investigate its functional effects on OC progression. ShRNA against KCNQ1OT1 was introduced into SKOV3 and SW626 cells, and the knockdown efficiency was confirmed via RT-qPCR assay, with sh-NC as negative control (Fig. 2A). Subsequently, a series of loss-of-function assays were carried out. MTT assay demonstrated that KCNQ1OT1 knockdown decreased the cell viability of OC cells (Fig. 2B). And less colonies were detected in sh-KCNQ1OT1 group compared to sh-NC group, as uncovered by colony formation assay (Fig. 2C). Wound healing assay and Transwell assay revealed that depletion of KCNQ1OT1 inhibited migration and invasion of SKOV3 and SW626 cells, respectively (Fig. 2D, E). Epithelial mesenchymal transition (EMT) is associated with tumorigenesis, and gives tumor cells migratory and invasive abilities (Tan et al. 2016). Additionally, EMT is characterized by the decreased expression of epithelial markers (including E-cadherin, α- and β-catenin) and increased expression of mesenchymal markers (including N-cadherin and vimentin). Here, Western blotting was applied for examining the levels of epithelial marker E-cadherin and mesenchymal marker N-cadherin, and results showed that KCNQ1OT1 deficiency increased E-cadherin level, while reduced N-cadherin level in OC cells (Fig. 2F). Taken together, KCNQ1OT1 knockdown repressed the proliferation and migration of OC.

### LncRNA KCNQ1OT1 could decrease EIF2B5 expression by promoting EIF2B5 methylation

**Fig. 3:**
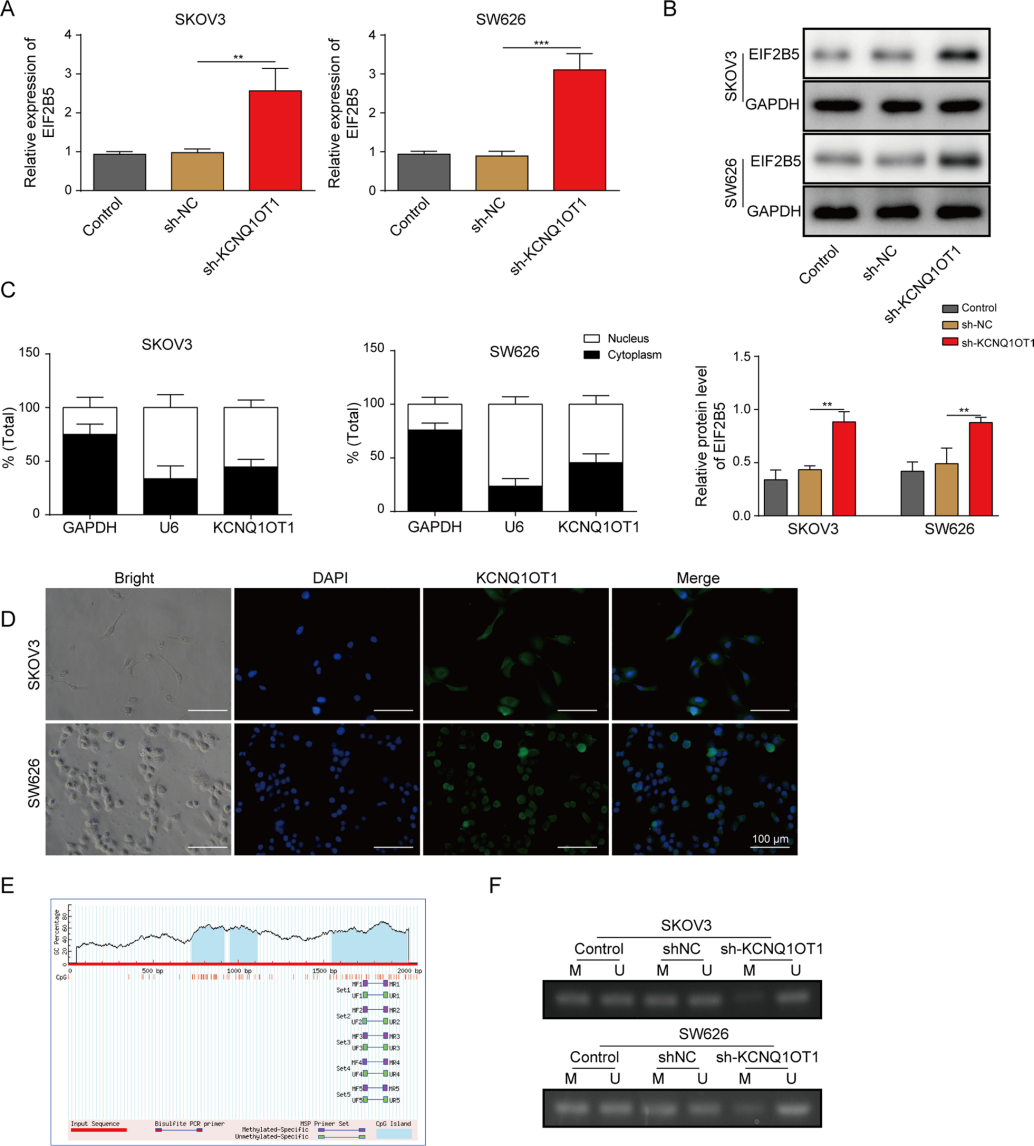
LncRNA KCNQ1OT1 could decrease EIF2B5 expression by promoting EIF2B5 methylation. **A**, **B** RT-qPCR analysis and Western blotting for the mRNA and protein levels of EIF2B5 in SKOV3 and SW626 cells transfected with Control (blank), sh-NC or sh-KCNQ1OT1. **C** Nuclear-cytoplasmic fractionation assay for the subcellular location of KCNQ1OT1 in SKOV3 and SW626 cells, GAPDH and U6 snRNA serving as internal control for cytoplasmic and nuclear cytoplasmic and nuclear fractions, respectively. **D** FISH analysis for the subcellular location of KCNQ1OT1 in SKOV3 and SW626 cells. **E** Prediction for the CpG island in EIF2B5 promoter region by MethPrimer 2.0. **F** MS-PCR assay for the methylation status of EIF2B5 promoter in SKOV3 and SW626 cells transfected with Control (blank), sh-NC or sh-KCNQ1OT1; M: Methylated primers, U: unmethylated primers. *P < 0.05

We then evaluated the influence of KCNQ1OT1 on EIF2B5 expression in OC cells. As illustrated in Fig. 3A, B, silencing of KCNQ1OT1 increased EIF2B5 expression in SKOV3 and SW626 cells. Data of Nuclear-cytoplasmic fractionation experiment and FISH analysis showed lncRNA KCNQ1OT1 located in nucleus and cytoplasm of SKOV3 and SW626 cells, and mainly in nucleus (Fig. 3C, D). Later, the mechanistic way of KCNQ1OT1 affected the expression of EIF2B5 was explored in SKOV3 and SW626 cells. MethPrimer 2.0 (http://www.urogene.org/methprimer2/tester-invitation.html) predicted that there existed CpG islands in the EIF2B5 promoter region (Fig. 3E). Furthermore, MS-PCR manifested that KCNQ1OT1 knockdown strikingly decreased the methylation level of EIF2B5 in SKOV3 and SW626 cells, in comparison with that in cell transfected with sh-NC (Fig. 3F). In sum, lncRNA KCNQ1OT1 could reduce EIF2B5 expression level by promoting EIF2B5 methylation.

### LncRNA KCNQ1OT1 increased EIF2B5 promoter methylation by recruiting DNA methyltransferases into its promoter region

**Fig. 4:**
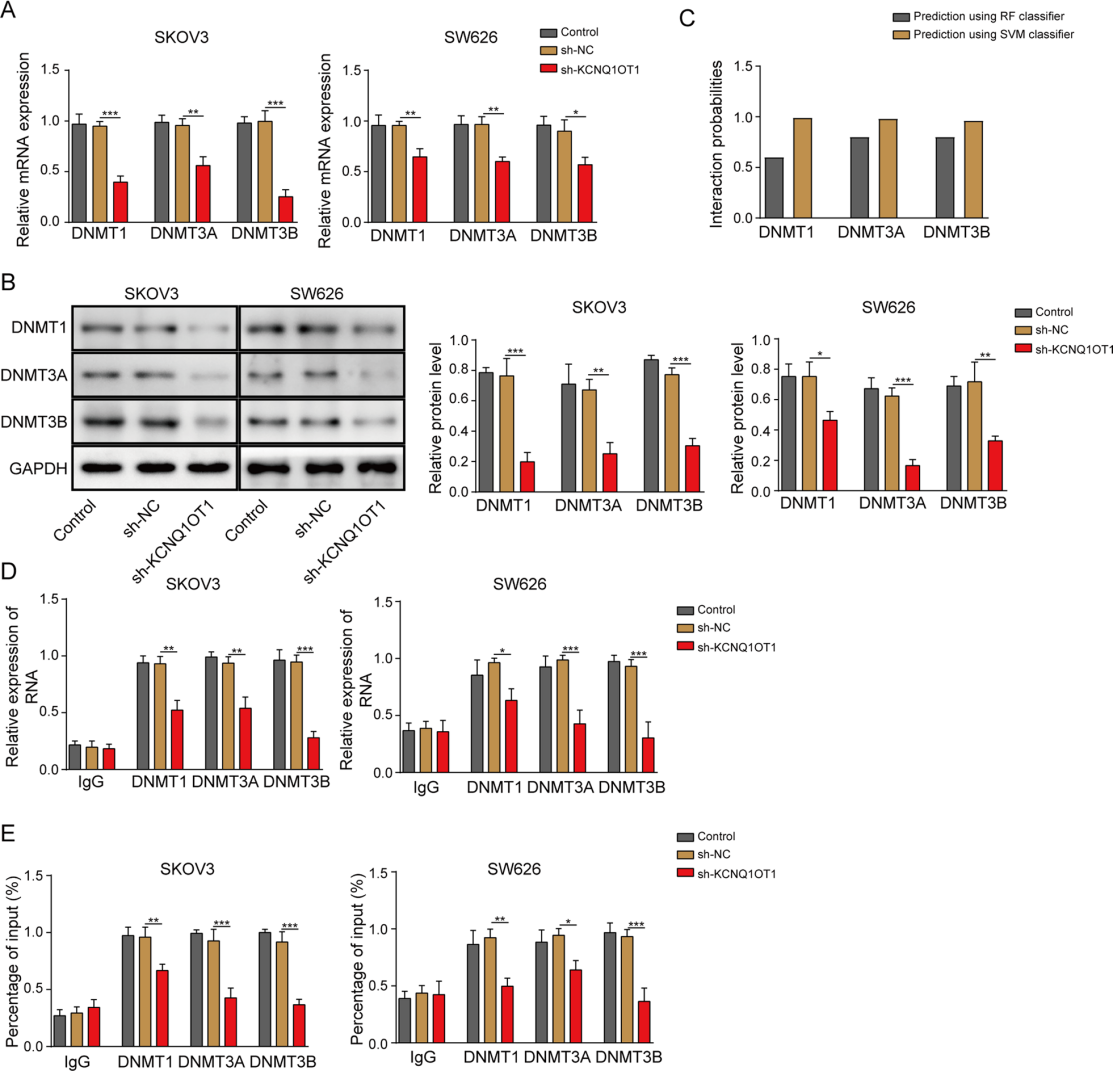
LncRNA KCNQ1OT1 increased EIF2B5 promoter methylation by recruiting DNA methyltransferases into its promoter region. **A**, **B** RT-qPCR analysis and Western blotting for the mRNA and protein levels of DNMT1, DNMT3A and DNMT3B in SKOV3 and SW626 cells transfected with Control (blank), sh-NC or sh-KCNQ1OT1. **C** Prediction for the binding potency between KCNQ1OT1 and DNMT1, DNMT3A or DNMT3B. **D** RIP assay for the combining ability of KCNQ1OT1 to DNMT1, DNMT3A and DNMT3B. **E** ChIP assay for the enrichment of DNMT1, DNMT3A and DNMT3B in EIF2B5 promoter. *P < 0.05

In order to further explore the regulatory mechanism between KCNQ1OT1 and EIF2B5, we analyzed the impact of KCNQ1OT1 on enrichment of DNA methyltransferases (DNMT1, DNMT3A and DNMT3B). As exhibited in Fig. 4A, B, KCNQ1OT1 deficiency remarkably decreased levels of DNMT1, DNMT3A and DNMT3B in SKOV3 and SW626 cells. Furthermore, RPIseq database showed a combining potential between KCNQ1OT1 and DNMT1, DNMT3A as well as DNMT3B, with interaction probabilities exceeding 0.5 (Fig. 4C). Following RIP assay uncovered that KCNQ1OT1 knockdown resulted in a significantly declined enrichment of DNMT1, DNMT3A and DNMT3B (Fig. 4D). Additionally, CHIP manifested that abundance of DNMT1, DNMT3A and DNMT3B was decreased in EIF2B5 promoter due to KCNQ1OT1 downregulation (Fig. 4E). Collectively, KCNQ1OT1 negatively regulated EIF2B5 expression via recruiting DNA methyltransferases into EIF2B5 promoter.

### EIF2B5 inhibition largely relieved the inhibitory impact of KCNQ1OT1 depletion on the malignant behaviors of OC cells

**Fig. 5:**
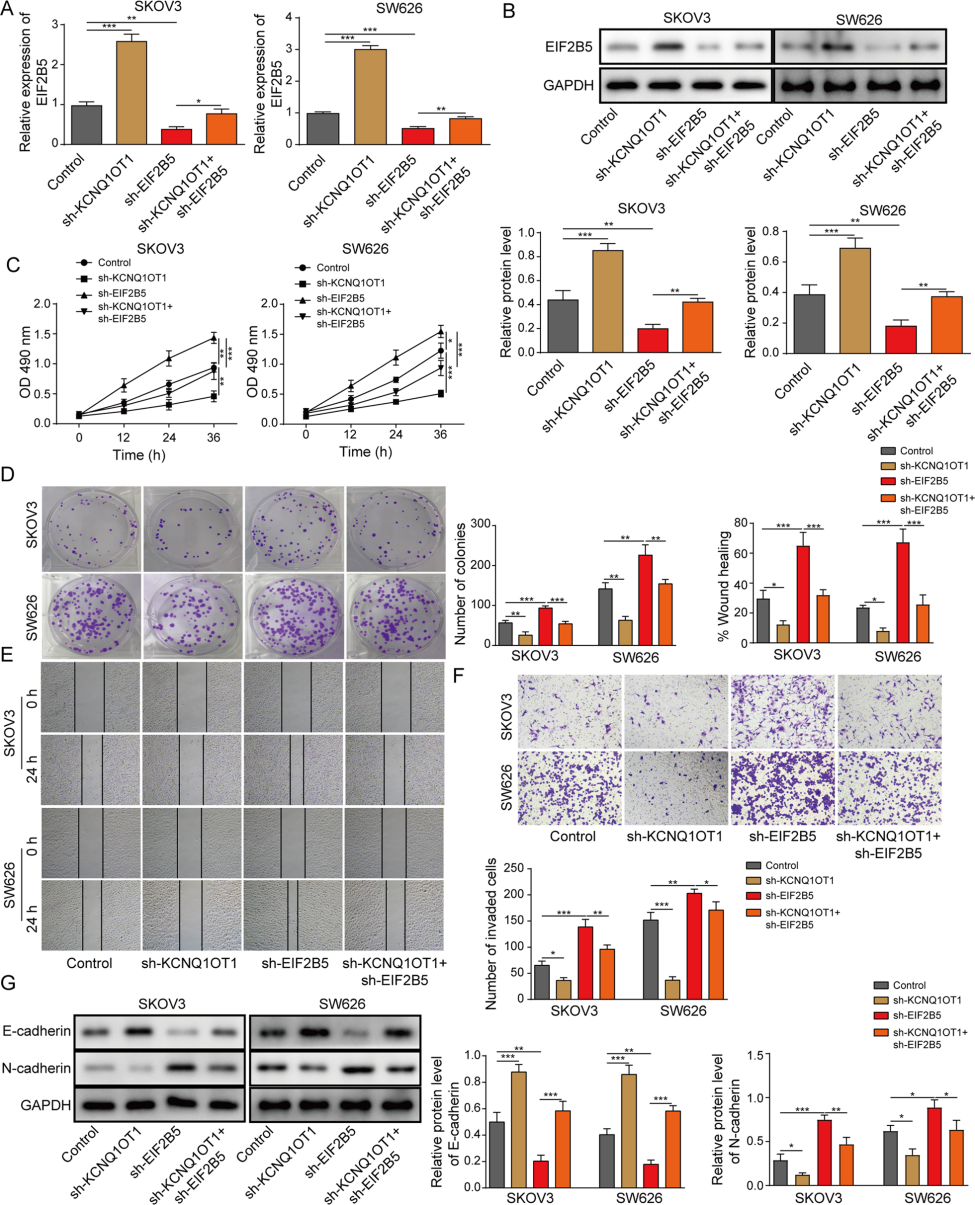
EIF2B5 inhibition largely relieved the inhibitory impact of KCNQ1OT1 depletion on the malignant behaviors of OC cells. SKOV3 and SW626 cells were transfected with Control (blank), sh-KCNQ1OT1, sh-EIF2B5 or sh-KCNQ1OT1 + sh-EIF2B5. **A**, **B** RT-qPCR analysis and Western blotting for the mRNA and protein levels of EIF2B5 in transfected cells. **C** MTT assay for the cell viability of transfected cells. **D** Colony formation assay for the cell clonogenicity capacity of transfected cells. **E** Wound healing assay for the cell migration of transfected cells. **F** Transwell assay for the cell invasion of transfected cells. **G** Western blotting for abundance of E-cadherin and N-cadherin proteins in transfected cells. *P < 0.05

To study whether the role of KCNQ1OT1 in OC progression was attributed to EIF2B5, shRNAs against KCNQ1OT1 and EIF2B5 were co-transfected into SKOV3 and SW626 cells. We found that the KCNQ1OT1 depletion-induced the increased EIF2B5 level in SKOV3 and SW626 cells was attenuated by EIF2B5 inhibition (Fig. 5A, B). In addition, silencing of KCNQ1OT1-induced the declined cell viability (Fig. 5C), clonogenicity (Fig. 5D), migration and invasion (Fig. 5E, F), as well as the elevated E-cadherin level and decreased N-cadherin level (Fig. 5G) in OC cells were all almost rescued by EIF2B5 inhibition. To sum up, KCNQ1OT1 knockdown inhibited OC progression by increasing EIF2B5 expression.

## Discussion

OC induces the highest mortality among malignancies derived from female reproductive system, resulting in approximately 1.5 × 10^5^ deaths annually (Permuth-Wey and Sellers 2009; Torre et al. 2018). Risk factors of OC include delivery, OC family history, body mass index (BMI) and smoking, of which BMI and smoking are closely associated with aggressive and fatal OC (Fortner et al. 2019). While, the mechanism of OC occurrence and development remains to be further explored. In this study, we detected the obvious upregulation of lncRNA KCNQ1OT1 in OC. Furthermore, we were the first to manifest that KCNQ1OT1 could contribute to the malignant properties of OC cells by downregulating EIF2B5 expression.

LncRNAs could perform as efficient biomarkers for OC diagnosis and prognosis clinically, and certain lncRNAs have potential to serve as molecular therapy targets (Zhong et al. 2016). Also, lncRNAs were implicated with OC acquired chemoresistance to platinum- and taxane-based medicine (Elsayed et al. 2020). LncRNA KCNQ1OT1 was multifunctional in human diseases, including tumors. For example, KCNQ1OT1 depletion could suppress cardiac hypertrophy by sponging miR-2054 and decreasing AKT3 expression (Chen et al. 2020a). KCNQ1OT1 promoted atherosclerosis development by modulating miR-452-3p/HDAC3/ABCA1 pathway (Yu et al. 2020). Additionally, KCNQ1OT1 exerted tumor-promoting roles in colorectal carcinogenesis (Chen et al. 2020b), non-small-cell lung carcinoma (Dong et al. 2019), hepatocellular carcinoma (Xu et al. 2020), bladder cancer (Wang et al. 2019a), glioma (Ding et al. 2020), osteosarcoma (Wang et al. 2019b), gastric cancer (Wang et al. 2020) and OC (Liu et al. 2020). Consistent to research of Liu et al. (2020), we found that KCNQ1OT1 expression was increased in OC tissues and cells. Moreover, our data also suggested that KCNQ1OT1 could positively regulate growth, metastasis and EMT of OC cells. Collectively, KCNQ1OT1 functioned as an oncogenic factor in human tumors.

EIF2B5 was disclosed to modulate the initial stage of protein synthesis, that could expedite angiogenesis and tumor growth (Fogli and Boespflug-Tanguy 2006). Enhanced EIF2B5 expression could indicate poor prognosis of liver cancer patients (Jiao et al. 2018). High EIF2B5 expression was associated with worse survival of colorectal cancer patients (Palaniappan et al. 2016). Furthermore, depletion of EIF2B5 facilitated MYC-driven apoptosis of colorectal cancer SW480 cells (Schmidt et al. 2019). Hou et al. demonstrated that EIF2B5 was lowly expressed in OC tissues (Hou et al. 2020). In this work, we also identified the downregulation of EIF2B5 in OC tissues and cells. And we firstly found that silencing of KCNQ1OT1 promoted EIF2B5 expression in OC cells.

We then further explored the mechanism of KCNQ1OT1 affecting EIF2B5 expression in OC cells. Nuclear-cytoplasmic fractionation assay revealed the main location of KCNQ1OT1 in nucleus of OC cells, suggesting that KCNQ1OT1 might participate in transcription regulation. LncRNAs were proved to alter DNA methylation, thereby impacting the expression of genes (Gao et al. 2019; Yu et al. 2019; Zheng et al. 2020). Hence, we wondered that KCNQ1OT1 might affect methylation of EIF2B promoter. Our experiments showed that KCNQ1OT1 depletion inhibited EIF2B5 methylation, and KCNQ1OT1 could bind with DNA methyltransferases, DNMT1, DNMT3A and DNMT3B. To sum up, KCNQ1OT1 could recruit DNA methyltransferases into EIF2B5 promoter, thereby negatively regulating EIF2B5 expression. Functionally, interference of EIF2B5 almost rescued KCNQ1OT1 depletion-induced repressed OC cell proliferation and metastasis. Actually, partial silencing of KCNQ1OT1 inhibited OC progression and metastasis, suggesting its important role in OC development. However, there might be other downstream gene of KCNQ1OT1, which might be responsible for functional effect of KCNQ1OT1 on OC development.

## Conclusion

LncRNA KCNQ1OT1 could promote OC growth and metastasis by increasing EIF2B5 methylation, thus downregulating EIF2B5 expression. The present study highlighted a novel molecular mechanism for OC development and the potential application of KCNQ1OT1 for OC treatment.

## Data Availability

All data generated or analyzed during this study are included in this article. The datasets used and/or analyzed during the current study are available from the corresponding author on reasonable request.

## Abbreviations

ANOVA: Analysis of variance
BCA: Bicinchoninic acid
cDNA: Complementary
CASC9: Cancer susceptibility 9
ChIP: Chromatin immunoprecipitation
DMSO: Dimethyl sulfoxide solution
ECL: Enhanced chemiluminescence
EIF2B5: Eukaryotic translation initiation factor 2B subunit 5
EMT: Epithelial–mesenchymal transition
FBS: Fetal bovine serum
HOTAIR: Homeobox transcript antisense intergenic RNA
KCNQ1OT1: KCNQ1 opposite strand/antisense transcript 1
lncRNAs: Long non-coding RNAs
MS-PCR: Methylation-specific PCR
MTT: 3-(4,5-Dimethyl-2-thiazolyl)-2, 5-diphenyl-2-*H*-tetrazolium bromide
NEAT1: Nuclear enriched abundant transcript 1
OC: Ovarian cancer
PBS: Phosphate buffered saline
PVDF: Polyvinylidene difluoride
RIPA: Radio-Immunoprecipitation Assay
RIP: RNA immunoprecipitation
RPMI-1640: Roswell Park Memorial Institute 1640 medium
SD: Standard deviations
SDS-PAGE: Sodium dodecyl sulfonate-polyacrylamide gel electrophoresis
